# Peg-in-Hole Assembly Based on Two-phase Scheme and F/T Sensor for Dual-arm Robot

**DOI:** 10.3390/s17092004

**Published:** 2017-09-01

**Authors:** Xianmin Zhang, Yanglong Zheng, Jun Ota, Yanjiang Huang

**Affiliations:** 1 Guangdong Provincial Key Laboratory of Precision Equipment and Manufacturing Technology, South China University of Technology, Guangzhou 510640, China; zhangxm@scut.edu.cn (X.Z.); mezhengyl1129@mail.scut.edu.cn (Y.Z.); 2 School of Mechanical and Automotive Engineering, South China University of Technology, Guangzhou 510640, China; 3 Research into Artifacts, Center for Engineering, The University of Tokyo, Chiba 113-8654, Japan; ota@race.u-tokyo.ac.jp

**Keywords:** peg-in-hole assembly, assembly scheme, force/torque sensor, dual-arm coordination, Baxter robot

## Abstract

This paper focuses on peg-in-hole assembly based on a two-phase scheme and force/torque sensor (F/T sensor) for a compliant dual-arm robot, the Baxter robot. The coordinated operations of human beings in assembly applications are applied to the behaviors of the robot. A two-phase assembly scheme is proposed to overcome the inaccurate positioning of the compliant dual-arm robot. The position and orientation of assembly pieces are adjusted respectively in an active compliant manner according to the forces and torques derived by a six degrees-of-freedom (6-DOF) F/T sensor. Experiments are conducted to verify the effectiveness and efficiency of the proposed assembly scheme. The performances of the dual-arm robot are consistent with those of human beings in the peg-in-hole assembly process. The peg and hole with 0.5 mm clearance for round pieces and square pieces can be assembled successfully.

## 1. Introduction

In the past few years, focus on compliant dual-arm robots has increased due to their safety and human-like manipulation [1,2,3,4]., Compliant dual-arm robots are often limited to low positioning accuracy, and thus usually applied to complete a daily task [4,5] or an industrial task that does not require high positioning accuracy [6,7]. In order to extend a compliant dual-arm robot to complete a precision task, such as precision peg-in-hole assembly, a reasonable assembling strategy and sensor information are required. Since the assembly pieces contact with each other in the process of assembly, the force information between the assembly pieces is usually taken into account in completing the peg-in-hole assembly tasks. Therefore, it is important to consider the force information, assembly strategy, and dual-arm manipulation when using a compliant dual-arm robot to complete a peg-in-hole assembly task.

In previous studies, many research studies focused on methods such as passive compliant assembly and active compliant assembly. For passive compliant assembly, Makino [8] designed a SCARA (selective compliance assembly robot arm) manipulator for assembly tasks, which has good compliance in the horizontal direction and large stiffness in the vertical direction. Whitney [9] developed a spring compliant mechanism named RCC (remote compliance center) to get a different compliance index and eliminate most of the stiffness error by adjusting the elasticity and angle of each spring. Many kinds of mechanisms have been designed for different applications based on the work of the RCC. Knight [10] presented a wrist mechanism called CVHRCC (Chamferless vertical/horizontal remote center compliance), which can accommodate position errors between assembly pieces. Even though spring and damping parts have been used to absorb and store energy produced by the interacting forces, forces information cannot be feedback to guide the motion of robot. On the other hand, many researchers paid attention to active compliant assembly [11,12,13,14,15,16,17,18,19]. Takahashi proposed a method for a robot to form interpretation rules by analyzing the human assembly operation [11]. Different assembly states were classified, and three kinds of shapes were taken to verify the usefulness of this method. Fei [12] analyzed the screw theory towards contact forces in three dimensions, and completed multiple triple peg-in-hole assembly. Song [13] proposed a shape recognition algorithm based on a six-axis F/T sensor, and a hole detection algorithm to recognize the peg and the hole. He also proposed a peg-in-hole strategy for complex-shaped parts based on a guidance algorithm [14]. Park [15] presented an intuitive assembly strategy inspired from human behaviors for a single arm to realize a square-shaped peg-in-hole assembly. Position identification in force-guided robotic assembly tasks was addressed and experiments were carried out on a lightweight robot to verify the effectiveness of the strategy [16]. In [17], a recognition model for different contact states was established and experiments were carried on Cartesian coordinate robot to verify the validity of proposed assembly algorithm. In [18], a generic algorithm was presented and position/orientation errors were compensated by Cartesian impedance control, which were applied to connect the charging plug with an automobile. Based on the analysis of contact states and forces, fuzzy force control strategies were used to complete dual peg-in-hole assembly [19]. In addition, a lightweight robot completes peg-in-hole assembly processes for flexible rubber objects using a Gaussian mixtures model scheme [20]. The contact models based on force guidance were introduced in [21]. In [22,23,24,25], some researchers focused on the avoidance of jamming and wedging to obtain the assembly conditions. Recently, some researchers focused on the peg-in-hole assembly by using a dual-arm robot due to the human-like coordinated operation [26,27,28,29,30]. A programmed passive compliance system and intuitive strategy were proposed for a dual-manipulator to complete a peg-in-hole assembly task [26]. Motion planning and interaction control during the dual-arm operation were considered [27]. Shauri used vision sensors and force sensors to estimate the pose of the assembly pieces and prevent excessive collision during the contact process [28]. Matthias introduced the flexible assembly applications completed by a dual-arm robot involving the human–robot cooperation [29,30].

In this paper, we aim to propose a two-phase scheme for a compliant dual-arm robot to complete a peg-in-hole assembly task. Real-time forces and torque information from a force/torque sensor (F/T sensor) are feedback that guide the position adjustment and orientation adjustment. A random search method is proposed in the outside position adjustment to search for the hole. Furthermore, we establish the corresponding models according to different contact situations, analyze the conditions of interaction force, and propose the orientation adjustment strategies in the orientation adjustment. To verify the effectiveness of proposed method, single-arm operation and dual-arm operation for round and square assembling pieces are conducted. Experiments prove that the proposed scheme is a feasible solution to realize the peg-in-hole assembly tasks, and that the dual-arm coordinated operation is more efficient than the single-arm operation. The contribution of this study is that we proposed a two-phase scheme for a compliant dual-arm robot to complete a peg-in-hole assembly task efficiently.

The problem formulation is described in Section 2. The proposed method is introduced in Section 3. The experiments and discussions are presented in Section 4, and the conclusion is summarized in Section 5.

## 2. Problem Formulation

**Figure 1:**
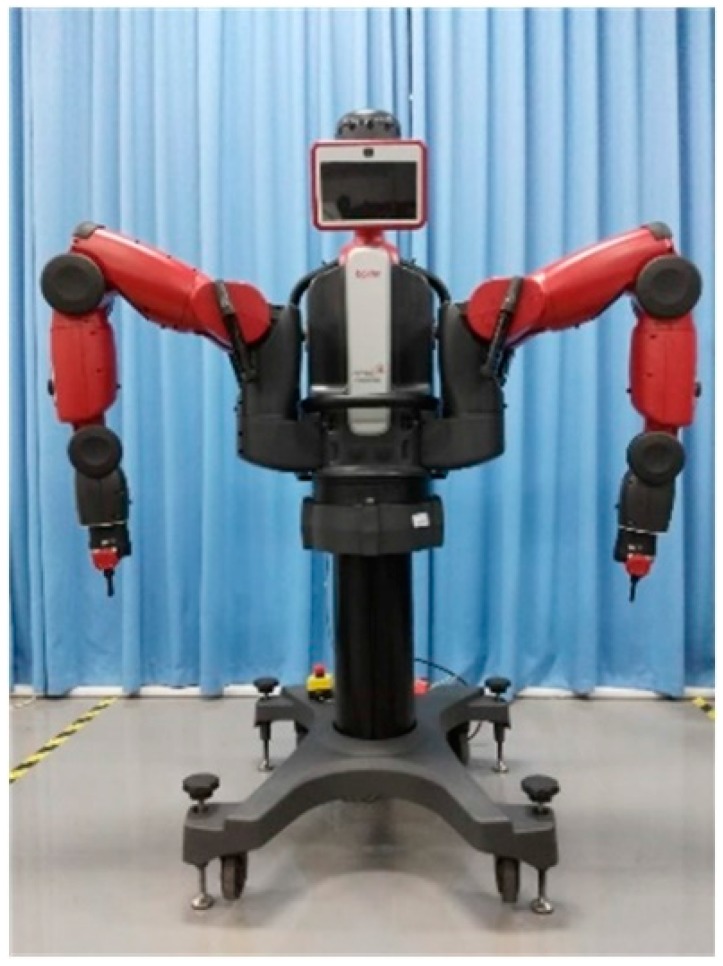
Baxter research robot.

**Figure 2:**
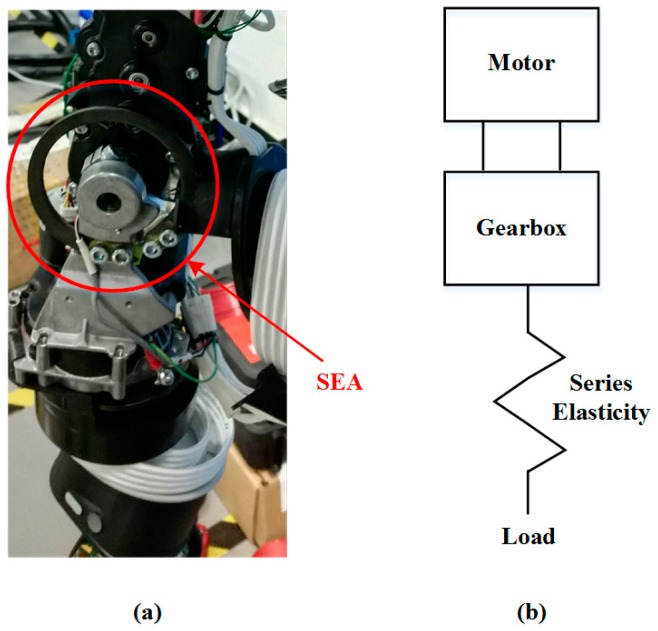
Series elastic actuators (SEA): (**a**) internal structure; (**b**) schematic.

In this study, we utilize the Baxter robot as the research platform [31], which is designed with seven degrees-of-freedom (7-DOF) on each arm to provide kinematic redundancy, as shown in Figure 1. The robot can be controlled and programmed by using a robot operating system (ROS) through the Baxter software developers kit (SDK) [32]. All of the joints are driven by series elastic actuators (SEA) [33], which differ from traditional actuation techniques in that they introduce a spring between the motor/gearing elements and the output of the actuator. The internal structure and schematic are illustrated in Figure 2. It makes the joints possess the characteristic of high flexibility, which brings the robot gains in stability and protection against shock loads. Thus, it is significant for us to apply such a high compliant robot into assembly applications. However, the following two difficulties need to be solved when using the compliant dual-arm robot to complete a peg-in-hole task:

1. Due to the introduction of springs and the transmission mechanism, nominal absolute positioning accuracy of the robot is low. How to realize a precise peg-in-hole assembly task with low positioning accuracy?
2. In order to adjust the interaction forces and torques within a certain short range, dual-arm coordinated operations need to be well realized.

Considering the above difficulties, we propose a two-phase assembly scheme that includes outside adjustment and inside adjustment to overcome the difficulties. A 6-DOF F/T sensor is installed on the right end-effector of the robot to detect the interaction forces and torques. Although the interaction forces and torques may be estimated from the sensor attached on each joint, the estimated forces and torques may be inaccurate due to the complex calculations and transformations. Therefore, we used a F/T sensor attached on the wrist to obtain the forces and torques information. The adjusting strategies of the robot are carried out according to the real-time force information. The proposed method is described in the next section.

## 3. Proposed Method

In this section, we will take round pieces assembly as an example to describe the proposed method and solve the problem formulated in Section 2. Inspired by the coordinated operation of human beings, we present a two-phase assembly scheme for a dual-arm robot to complete an assembly task in a human-like manner. Outside adjustment and inside adjustment are considered, respectively.

### 3.1. Assembly Behaviors

**Figure 3:**
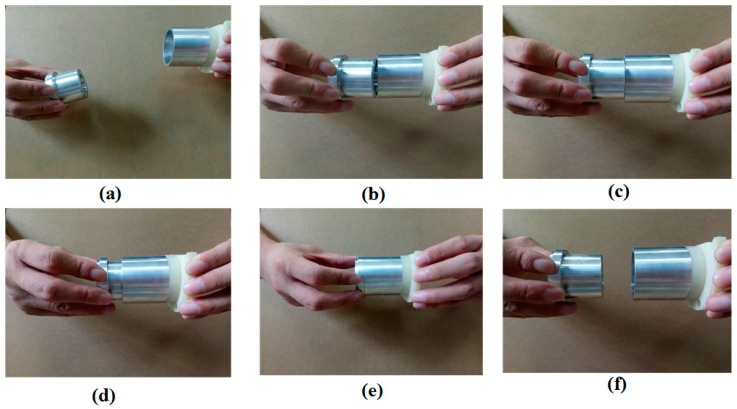
Human behaviors toward the peg-in-hole assembly based on contact information ((**a**): the peg approaches to the hole; (**b**) and (**c**): the peg and the hole are adjusting the orientation and position outside, respectively; (**d**) and (**e**): the peg and the hole are adjusting the orientation and position inside, respectively; (**f**): the peg removes from the hole).

Normally, a peg-in-hole assembly task can be completed by the dual-arm of a human being in a natural manner, as shown in Figure 3. The process of peg-in-hole assembly completed by a person can be described as follows: first, the person moves his arms roughly close to each other to make the peg contact the hole. Then, the person adjusts the orientations of both arms and randomly searches for the hole in a plane (perpendicular to the inserting direction) until the peg can insert into the hole. After that, the person adjusts the orientations of both arms to make the peg insert into the hole smoothly, until the peg inserts into the hole completely. At last, the peg is removed from the hole in the same way it was inserted.

**Figure 4:**
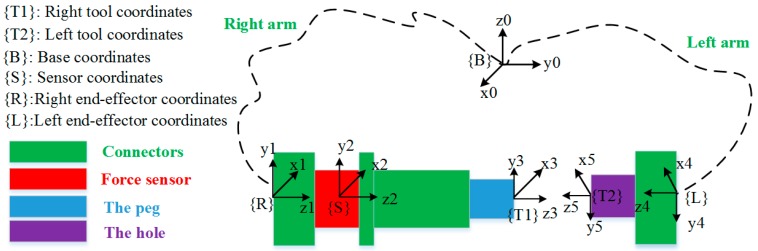
Transformation diagram of each coordinate frame.

Inspired by the assembly behaviors of human beings, we proposed an assembly strategy for a compliant dual-arm robot to complete a peg-in-hole assembly task in a human-like manner. We establish the relationship between the left arm and the right arm to realize the dual-arm coordinated operation. A transformation diagram of each coordinate frame is shown in Figure 4. Through the transformation between unit quaternion and rotation matrix expressed in {B}, real-time pose information of the end-effector coordinates of each dual-arm, {R} and {L}, are transformed to the tool coordinates, {T1} and {T2}, respectively. Besides this, the relationship between the sensor coordinate {S} and the right tool coordinate {T1} are also established. Thus, real-time force and torque information between {T1} and {T2} can be used as feedback information to guide the motion of the robot arms.

### 3.2. Two-Phase Assembly Scheme

**Figure 5:**
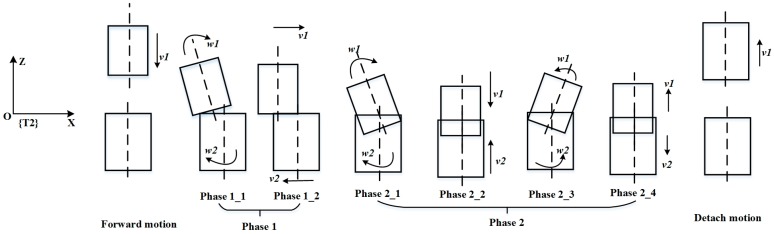
Two-phase assembly scheme (**Phase 1**: outside adjustment; **Phase 2**: inside adjustment; Phase 1_1 and Phase 1_2 are outside orientation adjustment and outside position adjustment, respectively; Phase 2_1, Phase 2_2, Phase 2_3 and Phase 2_4 are peg insertion orientation adjustment, peg insertion, peg removal orientation adjustment, and peg removal, respectively).

According to the human behaviors described in Section 3.1, a two-phase scheme is proposed in this paper, as shown in Figure 5. We describe the two-phase scheme using the left tool coordinate frame {T2} as the reference frame, which is located in the center of the end surface of the hole. The two-phase scheme includes an outside adjustment phase and an inside adjustment phase. From predefined initial positions of the dual-arm, the peg moves along the *Z*-axis at a certain step until the peg contacts the hole. Some misalignment between the assembly pieces is allowed. We define the outside adjustment phase as phase 1, which includes both outside orientation adjustment and outside position adjustment. Outside orientation adjustment is intended to make the peg and the hole aligned. During the outside position adjustment, the positions in the direction of the *Z*-axis are controlled to remain constant. A random search method is used to search for the hole within a small range of the *X–Y* plane. We define the inside adjustment phase as phase 2, which includes peg insertion orientation adjustment, peg insertion, peg removal orientation adjustment, and peg removal. Peg insertion orientation adjustment and peg removal orientation adjustment decrease the high torques resulted from high flexibility, and protect the assembly pieces from damage. Peg insertion and peg removal can accelerate the assembly process when torque is limited to a small value. To insert the peg into the hole smoothly, the torques around the *X*-axis and *Y*-axis are both required to be less than a threshold value in the process of assembly.

**Figure 6:**
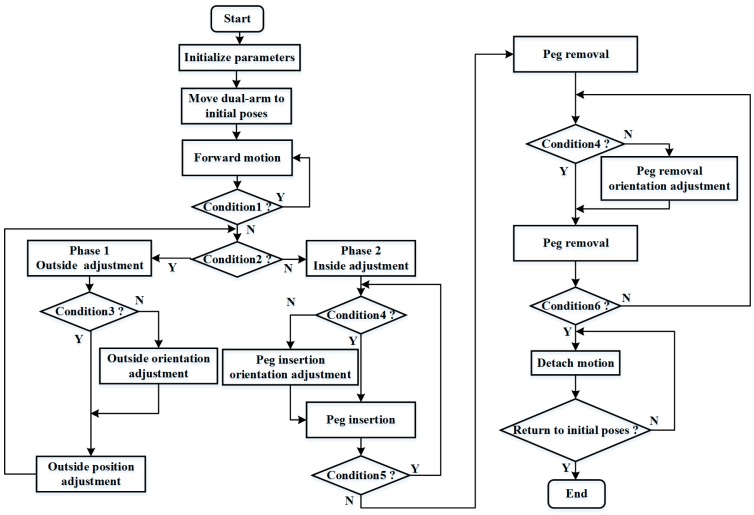
Control flow during the whole assembly process (Condition1: abs(Fz) < thresd_a; Condition2: diff_Fz < thresd_b; Condition3: abs(Mx) < thresd_d and abs(My) < thresd_e; Condition4: abs(Mx) < thresd_f and abs(My) < thresd_g; Condition5: abs(Fz) < thresd_c; Condition6: abs(Fz) < thresd_h and abs(Mx) < thresd_i and abs(My) < thresd_j).

Figure 6 shows the control flow during the whole assembly process. Real-time forces and torque information detected from the F/T sensor are required strictly to guide the motion of the robot. Once the assembly process starts, we initialize the parameters, including the values of the thresholds, inserting steps, initial poses, and moving speed of the dual-arm. The absolute value of *Fz* (i.e., force in the *Z*-axis direction) is regarded as the criterion of judgment between the adjacent phases. At first, the dual-arm is moved to the initial poses. Then, the peg approaches the hole at a certain step along the *Z*-axis of the right end-effector frame. When the absolute value of *Fz* exceeds a threshold value *thresd_a*, the peg is considered to contact the hole, and the two-phase scheme begins to work. The value of *diff_Fz* means the absolute difference of *Fz* between the adjacent time, as defined in Formula (1). If the absolute value of *Fz* exceeds *thresd_a* and *diff_Fz* is less than *thresd_b*, we consider the assembly process to be at phase 1. Otherwise, the assembly process is at phase 2. The peg inserts into the hole completely and begins to remove from the hole when the absolute value of *Fz* exceeds *thresd_c*. Besides, the absolute values of *Mx* (i.e., torque around the *X*-axis) and *My* (i.e., torque around the *Y*-axis) are both used as feedback information to judge the directions of rotation at the outside and inside orientation adjustment. At phase 1, if the absolute value of *Mx* and *My* are not less than *thresd_d* and *thresd_e*, respectively, we consider that the assembly process is at the outside orientation adjustment phase, and the orientations of the assembly pieces are adjusted to make the peg align with the hole. Otherwise, the assembly process is at the outside position adjustment phase, and the positions of the assembly pieces are adjusted to search for the hole. At phase 2, if the absolute values of *Mx* and *My* are not less than *thresd_f* and *thresd_g*, respectively, the orientations of the assembly pieces are adjusted to make the peg align to the hole during the peg insertion orientation adjustment and the peg removal orientation adjustment. Otherwise, the peg inserts into or removes from the hole at a certain step along the *Z*-axis of the right end-effector frame during peg insertion and the peg removal. When the peg is being removed from the hole and the absolute values of *Fx*, *Mx,* and *My* are less than *thresd_h*, *thresd_i* and *thresd_j*, respectively, the peg and the hole are considered to be separated. At last, the peg and the hole return to the initial poses by moving a certain step along the *Z*-axis of the right end-effector frame and left end-effector frame, respectively. When the dual-arm both return to the initial poses, the whole assembly process is completed.

*diff_Fz* = *Fz* (*t* + *Δt*) − *Fz* (*t*) *t* > 0 (1)

where, *Δt* is equal to the reciprocal of acquisition frequency.

### 3.3. Phase 1: Outside Adjustment

#### 3.3.1. Outside Position Adjustment

**Figure 7:**
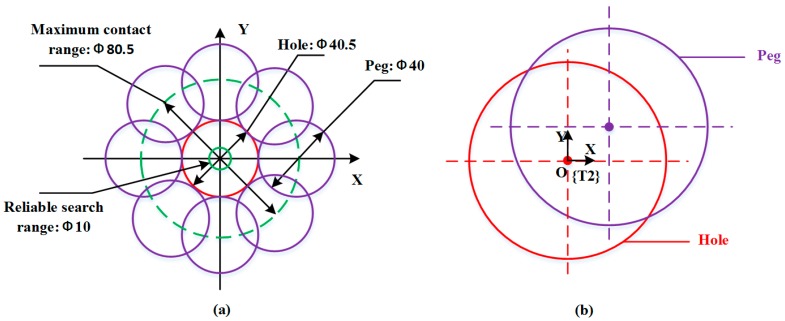
Position adjustment strategy: (**a**) a random search method; (**b**) dual-arm relative movement.

Due to the low positioning accuracy of the Baxter robot, a random search method is adopted to search for the hole in the outside position adjustment phase. As shown in Figure 7a, purple cycles refer to when the peg rotates along the edge of the hole. The hole is referred to as the red cycle. The green dotted cycle means the maximum contact range. A set of dual-arm positions appropriated for assembly is given in advance. According to our previous study on the positioning accuracy of the Baxter robot [6], the positioning accuracy of ±5 mm is taken into account. At each iteration, the peg searches for the hole in a reliable range, as shown in the green solid cycle in Figure 7a. The position and orientation in the direction of the *Z*-axis are both kept in a constant value, and a set of random positions in the *X-Y* plane are tested by adjusting the motion of the dual arms. At each iteration of random search, a guiding direction for the next random search is obtained to make the random search converge to the reasonable solution. The relative movements of the dual-arm along the *X*- and *Y*-axis are adopted to reduce the searching time and improve assembly efficiency. As shown in Figure 7b, if we take the left tool coordinate frame {T2} as the reference frame, the peg and the hole are moving in the relative directions along the *X*- and *Y*-axis when their center lines are not collinear. For example, the peg will move along the positive direction of the *X*-axis when the hole is moving along the negative direction of the *X*-axis.

#### 3.3.2. Outside Orientation Adjustment

High torques in three directions may result in clamping and wedging, which will make the assembly fail. The goal of orientation adjustment is to make the peg align with the hole in a way that reduces the torques. To reduce the forces and torques during the assembly process, the orientations of robot end-effectors should be adjusted in a reasonable direction. As shown in Table 1, we define the rotation directions of the dual arms according to the forces and torques information. The orientation adjustment for the dual-arm is determined based on the conditions of torque. For example, when the torque conditions are *Mx* > 0 and *My* > 0, the left arm should rotate in the positive direction around the *X*-axis, and rotate in the negative direction around the *Y*-axis. The right arm should rotate in the positive direction around the *X*-axis and *Y*-axis. We define the left response as the motion of the left robot arm according to the torque condition, and define the right response as the motion of the right robot arm according to the torque condition. All rotation directions are relative to the tool coordinate frames of the dual arms {T1} and {T2}. According to the torque conditions, we propose an orientation adjusting scheme, as shown in Table 2.

**Figure 8:**
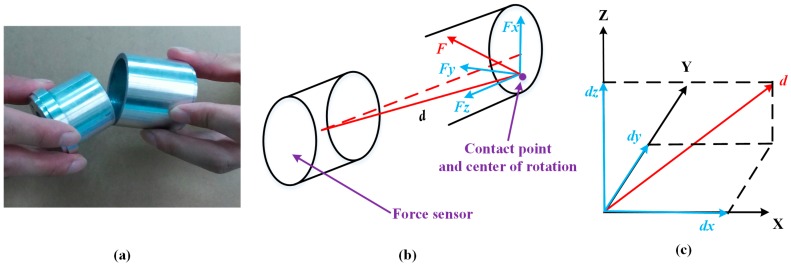
Outside of the single-point contact model: (**a**) contact situation; (**b**) force analysis and center of rotation; (**c**) displacement decomposition.

**Figure 9:**
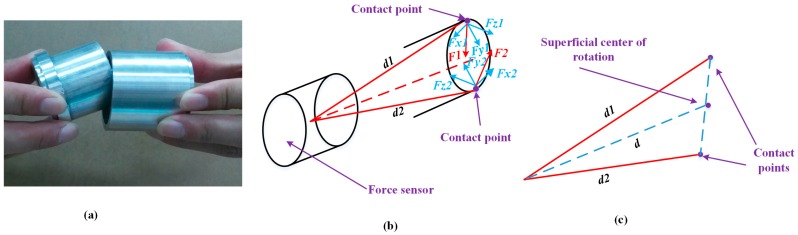
Outside of the two-point contact model: (**a**) contact situation; (**b**) force analysis and contact of rotation; (**c**) center of rotation.

At the outside orientation adjustment, there are two situations according to the different positions of the contact points. One is a single contact point on the end face of the peg, and the other situation involves two contact points on the end face of the peg, as shown in Figure 8 and Figure 9, respectively. In these two cases, an outside single-point contact model and an outside two-point contact model are established accordingly. In the single-point contact model, as shown in Figure 8, the contact point coincides with the center of rotation. The value of *d* represents the displacement from the center of the F/T sensor to the center of rotation. In the two-point contact model, as shown in Figure 9, the values of *d1* and *d2* represent the displacement from the center of the F/T sensor to the contact points. The center of rotation is simplified as the midpoint of the line formed by the two contact points. The force at the contact point and the displacement *d* can be decomposed along the three coordinate directions. The forces and torques detected from the F/T sensor are relevant due to the offset between the contact point and the center of F/T sensor, as shown in Equations (2) and (3). Based on the analysis of the two kinds of contact models, we simplistically identify the center of rotation, which can be calculated through Formula (4). Finally, the dual-arm rotates around the center of rotation to align the peg with the hole.

(2) $$\left\{ \begin{array}{l} F_{x}\cdot d=M_{y}\\ F_{y}\cdot d=M_{x} \end{array}\right.,$$

(3) $$\left\{ \begin{array}{l} F_{x1}\cdot d_{1}+F_{x2}\cdot d_{2}=M_{y}\\ F_{y1}\cdot d_{1}+F_{y2}\cdot d_{2}=M_{x} \end{array}\right.,$$

(4) $$\left\{ \begin{array}{l} M_{z}+F_{x}\cdot dy+F_{y}\cdot dx=0\\ M_{y}+F_{x}\cdot dz+F_{z}\cdot dx=0\\ M_{x}+F_{y}\cdot dz+F_{z}\cdot dy=0 \end{array}\right.$$

### 3.4. Phase 2: Inside Adjustment

**Figure 10:**
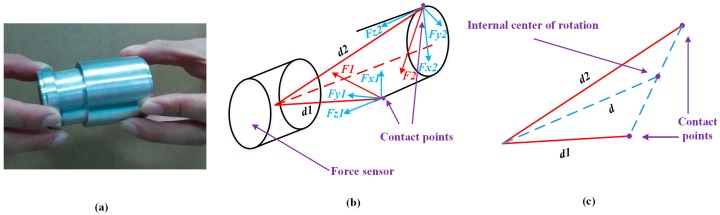
Inside of the two-point contact model: (**a**) contact situation; (**b**) force analysis and contact of rotation; (**c**) center of rotation.

Inside adjustment includes peg insertion orientation adjustment, peg insertion, peg removal orientation adjustment, and peg removal. There are two contact points in the peg insertion orientation adjustment and peg removal orientation adjustment. One is on the outer contour of the end face of the peg, and the other is on the rotation face of the peg, as shown in Figure 10. In this case, an inside two-point contact model is established, and the forces at the contact points are analyzed. The center of rotation is designated as the midpoint of the line formed by two contact points. Compared to the outside two-point contact model, the center of rotation in the inside two-point contact model locates in the interior of the peg. The theoretical rotation center can be calculated through Formula (4), and the adjusting scheme is the same as shown in Table 2. When the torques between the assembling pieces are adjusted into a reasonable range, the peg inserts into or removes from the hole with a certain step length. The inside orientation adjustment is done until the peg completely inserts into or removes from the hole.

## 4. Experiments

**Figure 11:**
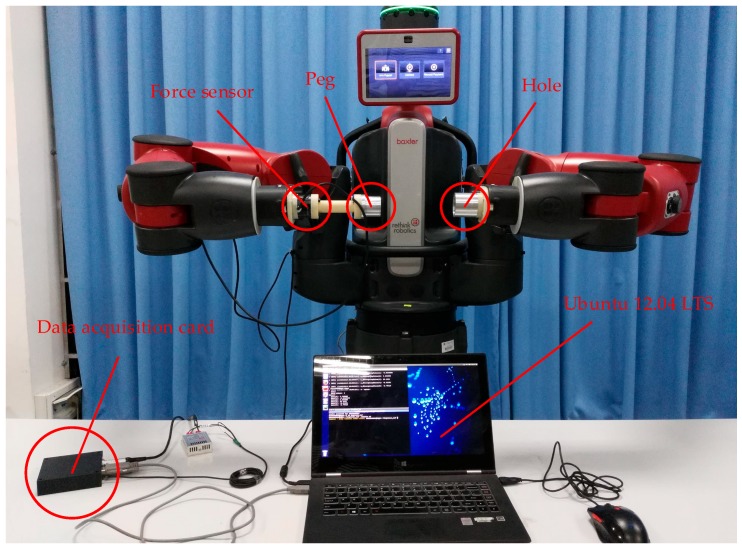
Experimental platform for a peg-in-hole assembly task.

In this section, experiments including single-arm operation and dual-arm operation for round pieces and square pieces were conducted to verify the effectiveness of the proposed two-phase assembly scheme. During the single-arm operation, we kept the left arm fixed, and made the right arm move to complete the assembly tasks. Figure 11 shows the experimental platform for the peg-in-hole assembly task. The F/T sensor (SRI M3813C) was attached on the end-effector of the right arm. The details about the F/T sensor can be found in [34]. The peg was attached to the F/T sensor with a connecting link. The hole was attached on the end-effector of the left arm. For round pieces and square pieces, the maximum clearance between the peg and the hole were 0.5 mm, and the length of the hole were 30 mm. The peg-in-hole assembly task includes the insertion of a peg into a hole, and its removal from the hole. The forces and torques information during the assembly process were derived through a data acquisition card. The control programs included a serial communication node written in C++ and a robot control node written in Python, which were developed through ROS with the Ubuntu 12.04 LTS system. The computing platform was based on an Intel (R) Core (TM) i5-4200U CPU @ 1.60 GHz, RAM 4 GB.

In the parameter setting for the Baxter robot and F/T sensor, the initial poses of the left arm and right arm were predefined: (0.70, 0.08, 0.30) and (0.70, −0.16, 0.30) were the initial positions for the end-effectors of the left arm and the right arm, respectively, and (0.076, −0.703, 0.704, 0.075) and (−0.028, −0.712, −0.701, 0.039) were the initial orientations for the end-effectors of the left arm and the right arm, respectively. The length that the peg moves along the *Z*-axis was set to 5 mm. The parameters for the robot moving are shown in Table 3. The frequency for data collection from the F/T sensor was set to 20 Hz. For the parameters in the assembly flow diagram, we set *thresd_a* = 4 N, *thresd_b* = 2 N, *thresd_c* = 4 N, *thresd_d* = *thresd_e* = 200 Nmm, *thresd_f* = *thresd_g* = 400 Nmm, *thresd_h* = 0.5 N, *thresd_i* = *thresd_j* = 20 Nmm. In this study, the threshold values were determined based on the identification of the force sensor, and the recognition of the robot motion with the force sensor.

### 4.1. Peg-in-Hole Assembly by Single Arm

**Figure 12:**
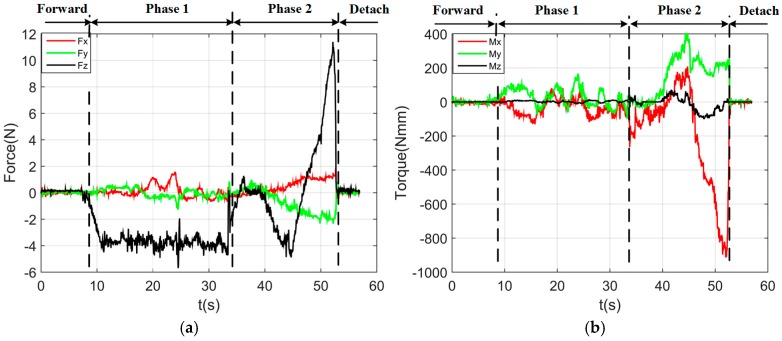
Round pieces: interacting forces and torques toward single-arm operation (Phase 1: outside adjustment phase; Phase 2: inside adjustment phase). (**a**) Interaction forces; (**b**) interaction torques.

**Figure 13:**
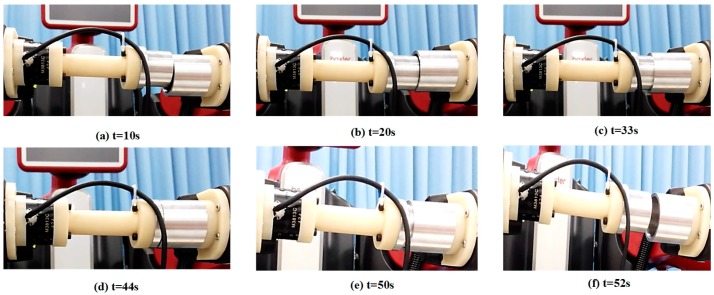
Round pieces: the process of peg-in-hole assembly with single-arm operation.

**Figure 14:**
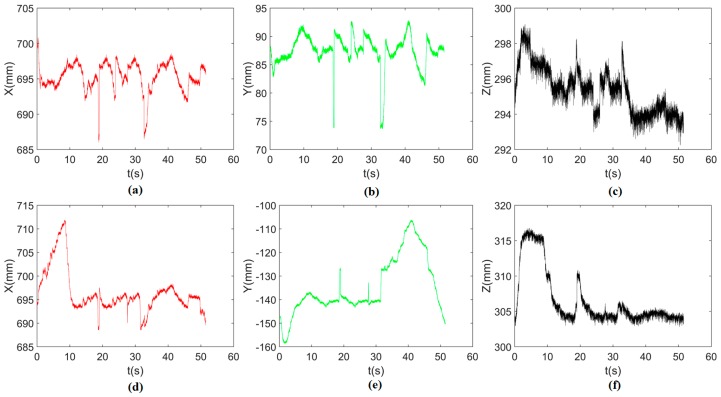
Round pieces: positions in three directions during the single-arm operation ((**a**–**c**) represent the positions of the left end-effector along the *X*-, *Y*- and *Z*-axis, respectively; (**d**–**f**) represent the positions of the right end-effector along the *X*-, *Y*- and *Z*-axis, respectively; the reference coordinate frame is the robot base coordinate frame).

**Figure 15:**
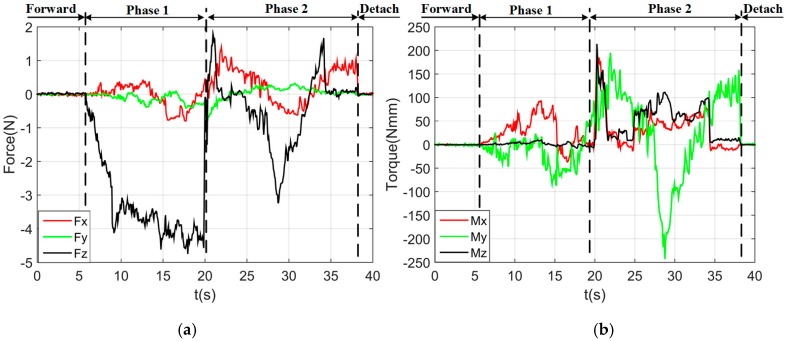
Square pieces: interacting forces and torques toward single-arm operation (Phase 1: outside adjustment phase; Phase 2: inside adjustment phase). (**a**) Interaction forces; (**b**) interaction torques.

By using the single arm to complete the peg-in-hole assembly task, we took the right arm with the peg as the adjusting arm, and the left arm with the hole remained fixed. In the peg-in-hole assembly for the round pieces, the interacting forces and torques were shown in Figure 12a,b, respectively. During the forward and detach motions, all of the forces and torques are close to 0 because the peg does not contact the hole. At the start of the outside adjustment phase, the torques had a little change, but the *Fz* increased to −4 N gradually when the peg was squeezing the hole. *Mx* and *My* were limited within ±200 Nmm, and *Fx* and *Fy* were also limited within small values. The small forces and torques in the *X*-axis and *Y*-axis can make the peg smoothly search for the hole. Since the positions in the direction of the *Z*-axis were fixed, *Fz* is slightly fluctuated near −4 N. At the start of the inside adjustment phase, the peg inserted into the hole until *Fz* reached −4 N. *Mx* and *My* were limited within ±400 Nmm. The experimental results showed that the proposed two-phase assembly scheme can make the robot complete a peg-in-hole assembly task with a single-arm operation. In this experiment, the peg-in-hole assembly with single-arm operation cost 55 s. The process of assembly with single-arm operation is shown in Figure 13. The trajectories of the left end-effector and the right end-effector under the single-arm operation are shown in Figure 14. Due to the compliance of the robot, the trajectory has some fluctuation when the peg contacts the hole. However, the peg can be inserted into the hole based on the proposed assembly scheme. Figure 15 showed the interaction forces and torques for the square peg-in-hole assembly under the single-arm operation. Compared with round pieces, the *M*z of square pieces is a relatively large value without considering the orientation of the *Z*-axis during the inside adjustment phase. However, due to the compliance of the dual-arm robot, the peg-in-hole assembly with square pieces can be completed by the single-arm operation under the proposed assembly scheme.

### 4.2. Peg-in-Hole Assembly by Dual-Arm

**Figure 16:**
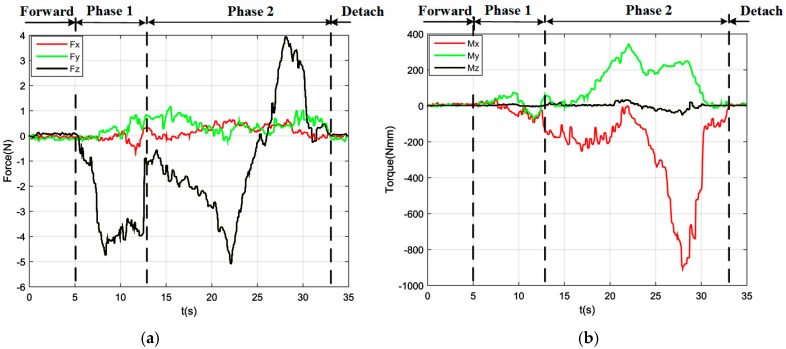
Round pieces: interaction forces and torques toward dual-arm operation (Phase 1: outside adjustment phase; Phase 2: inside adjustment phase). (**a**) Interaction forces; (**b**) interaction torques.

**Figure 17:**
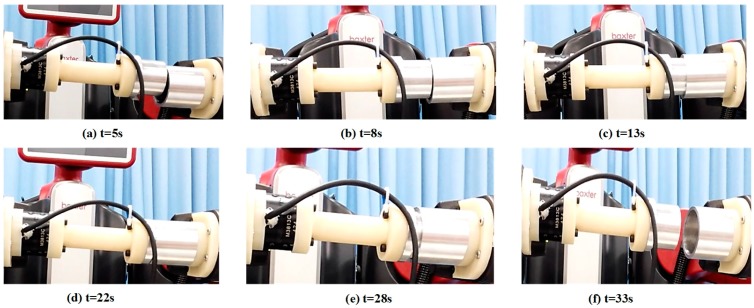
Round pieces: the process of peg-in-hole assembly with dual-arm operation.

**Figure 18:**
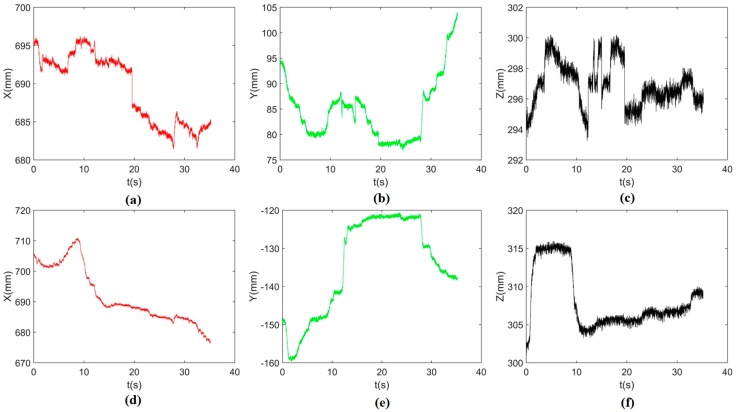
Round pieces: positions in three directions during the dual-arm operation ((**a**–**c**) represent the positions of the left end-effector along the *X*-, *Y*- and *Z*-axis, respectively; (**d**–**f**) represent the positions of the right end-effector along the *X*-, *Y*- and *Z*-axis, respectively; the reference coordinate frame is the robot base coordinate frame).

**Figure 19:**
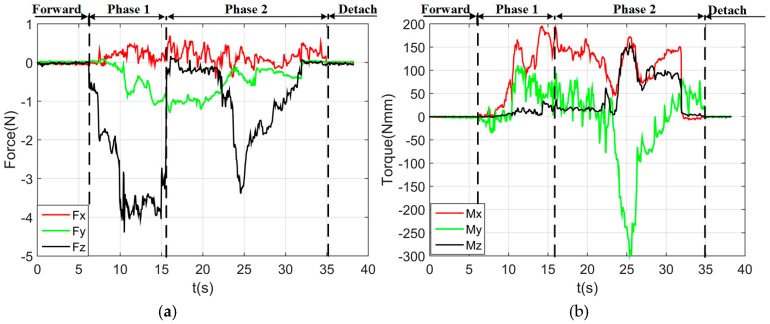
Square pieces: interacting forces and torques toward dual-arm operation (Phase 1: outside adjustment phase; Phase 2: inside adjustment phase). (**a**) Interaction forces; (**b**) interaction torques.

By using a dual-arm to complete the peg-in-hole assembly task, we made the dual arms realize the assembly as human beings would. In the peg-in-hole assembly for the round pieces, the interaction forces and torques are shown in Figure 16a,b, respectively. As shown in the results, *Fx* and *Fy* had no significant changes during the assembly process. *Fz* fluctuated between −6 N and +4 N. *Mx* was limited within ± 400 Nmm, except at the point when the peg touched the bottom face of the hole. In this experiment, the peg-in-hole assembly with dual-arm operation cost 35 s. The process of assembly with dual-arm operation is shown in Figure 17. From the experimental results, we can find that the two-phase scheme was feasible for the dual-arm operation to complete a peg-in-hole assembly task. The trajectories of the left end-effector and the right end-effector under the dual-arm operation were shown in Figure 18. From the results, we can find that the trajectory fluctuation for the dual-arm operation is smaller than that of the single-arm operation. This is because both arms are moving under the dual-arm operation. Even when the peg contacted the hole, the contact force was limited to a small value. Figure 19 showed the interaction forces and torques for the square peg-in-hole assembly under the dual-arm operation. The peg-in-hole assembly with square pieces can be completed by the dual-arm operation under the proposed assembly scheme. Due to space limitations, the assembly process and the trajectory of the robot for the square pieces assembly were not shown in this paper.

**Figure 20:**
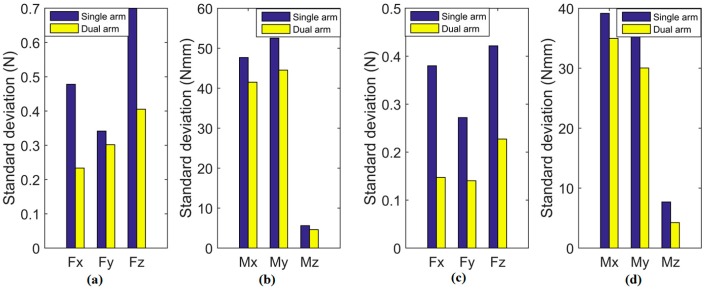
Comparison of forces and torques deviation ((**a**,**b**): deviation of forces and torques of round pieces, respectively; (**c**,**d**): deviation of forces and torques of square pieces, respectively).

**Figure 21:**
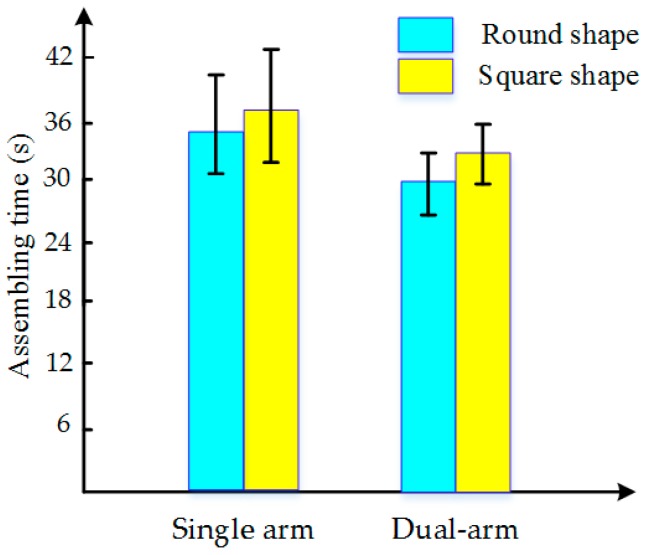
Assembling time for single-arm operation and dual-arm operation.

To evaluate the stability of the proposed method and the efficiency of dual-arm operation, round pieces and square pieces were both conducted 10 times in experiments toward the single-arm operation and dual-arm operation. Figure 20 shows the standard deviation of forces and torques, and Figure 21 shows the average assembling time in these experiments. For both round pieces and square pieces, the standard deviation of forces and torques derived by the dual-arm operation were smaller than that derived by the single-arm operation. This can make the peg insert into the hole smoothly without sudden change. Besides, average assembling time by using dual-arm operation is shorter than that using the single-arm operation. This is because the dual-arm operation adopts the principle of relative motion in the outside position adjustment phase, which can reduce the times of random searching. In the orientation adjustment phase, the dual-arm operation performs well due to the advantages of coordinated operation similar to human beings. One of the benefits from this coordinated operation is that the forces and torques in the dual-arm operation are limited to a reasonable range (as shown in Figure 16 and Figure 19), which makes the assembly smooth.

### 4.3. Peg-in-Hole Assembly by Human Beings

To verify the effectiveness of the proposed assembly scheme for a dual-arm robot, we compared the performance of the dual-arm robot to that of human beings through a peg-in-hole assembly task. The peg-in-hole assembly task was completed by five volunteers who did not have any knowledge of the force feedback mechanism in robotics. Each volunteer conducted the experiments 10 times. In order to recreate the experimental set-up of the robot, each volunteer held the hole in their left hand and the peg in their right hand. The F/T sensor connected with the peg was held by the right hand to extract the forces and torque values during the assembly process. An eye mask was also used to cover the eyes of the volunteers. The moving speed and moving step length were set to approximate the values as that for the robot. In the absence of vision, the volunteers needed to adjust the position and orientation of the assembling pieces based on the contact force, which coincides with the goal of this experiment.

**Figure 22:**
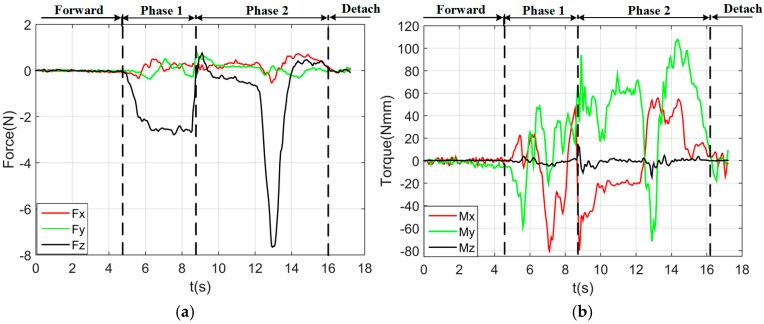
Round pieces: interaction forces and torques toward human beings (Phase 1: outside adjustment phase; Phase 2: inside adjustment phase). (**a**) Interaction forces; (**b**) interaction torques.

**Figure 23:**
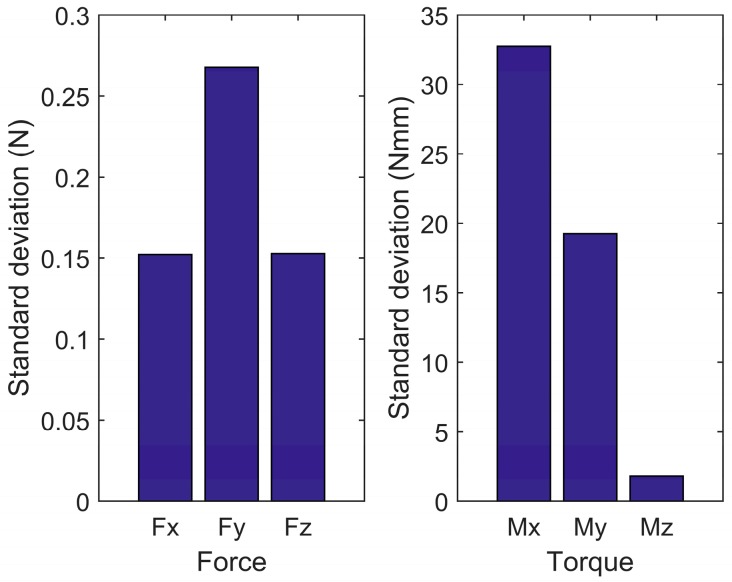
Forces and torques deviation of human beings.

**Figure 24:**
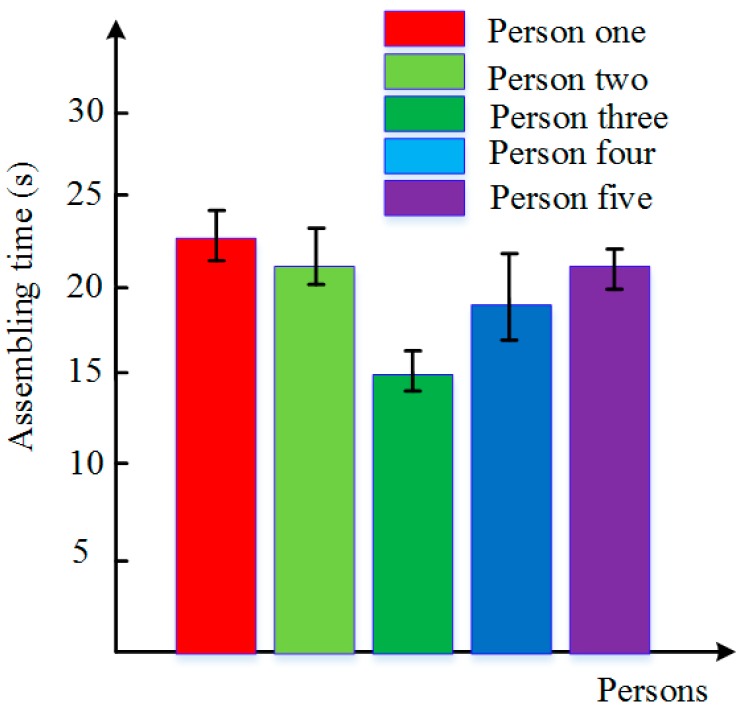
Assembling time for human beings.

As shown in Figure 22a,b, the changing trends of force and torque for the human assembly were similar to those for the robot assembly. During phase 1, the absolute value of Fz was getting bigger at first, until the volunteer felt that the peg and the hole were in contact with each other. The arms of volunteers began to adjust the orientation of the assembly pieces to make the peg align with the hole. Then, volunteers kept the assembly pieces in contact and then searched for the hole in a blind search method until the absolute value of Fz decreased suddenly, which means the peg inserted into the hole and began the inside adjustment. When the absolute value of Fz was increasing rapidly, the volunteer felt the peg inserted into the hole completely, and removed from the hole until the whole assembly process was completed. During the whole process, the torques along the *X*-and *Y*-axis were well controlled within 100 Nmm. Figure 23 showed the deviation of forces and torques during the outside adjustment phase. We can conclude that human beings have a better performance in adjusting the orientation than a robot. The average assembly time of five volunteers is shown in Figure 24. From these results, we know that human beings can complete the peg-in-hole assembly tasks in a short time, and find that the performance of the dual-arm robot was similar to that of human beings in a peg-in-hole assembly process.

## 5. Conclusions

In this paper, we proposed a two-phase assembly scheme for a compliant dual-arm robot to complete a peg-in-hole task using the feedback information of F/T sensor. The proposed assembly scheme was inspired by the performance of human beings in the peg-in-hole assembly process. The position and orientation of the assembling pieces were adjusted during the assembly process. Two contrast experiments consisting of a single-arm operation and a dual-arm operation were conducted to verify the effectiveness of the proposed scheme by assembling round pieces and square pieces. The peg and hole with 0.5 mm clearance could be assembled successfully. The dual-arm operation had better performance gain in controlling the forces and torques, which can effectively avoid the occurrence of jamming and wedging. Alongside this, the peg-in-hole assembly experiments completed by human beings were also conducted. The performance of the robot was consistent with the performances of human beings. From the experimental results, we can conclude that the two-phase scheme is applicable for a compliant robot to complete a peg-in-hole assembly task, and the dual-arm operation is more efficient than the single-arm operation.

In future work, the complex shapes of assembly pieces will be tested and vision recognition will be taken into account at the non-contact phase.

## Figures and Tables

**Table 1:** Relationship between the forces and torques.

Force Suffered	Torque Produced	Left Arm Responses	Right Arm Responses
*Fx > 0*	*My > 0*	*L (0*,*−1*,*0) ^1^*	*R (0*,*+1*,*0) ^2^*
*Fx < 0*	*My < 0*	*L (0*,*+1*,*0)*	*R (0*,*−1*,*0)*
*Fy > 0*	*Mx < 0*	*L (−1*,*0*,*0)*	*R (−1*,*0*,*0)*
*Fy < 0*	*Mx > 0*	*L (+1*,*0*,*0)*	*R (+1*,*0*,*0)*

*^1^ L(0*,*−1*,*0)* means that the left arm rotates in a negative direction around the *Y*-axis of sensor coordinate frame {S}; *^2^ R(0*,*+1*,*0)* means that the right arm rotates in a positive direction around the *Y*-axis of sensor coordinate frame {S}.

**Table 2:** Orientation adjusting scheme.

Torque Conditions	Left Responses	Right Responses
*Mx > 0 and My > 0*	*L (1*,*0*,*0) and L (0*,*−1*,*0) ^1^*	*R* *(1*,*0*,*0) and* *R* *(0*,*1*,*0) ^2^*
*Mx > 0 and My < 0*	*L (1*,*0*,*0) and L (0*,*1*,*0)*	*R* *(1*,*0*,*0) and* *R* *(0*,*−1*,*0)*
*Mx < 0 and My > 0*	*L (−1*,*0*,*0) and L (0*,*−1*,*0)*	*R* *(−1*,*0*,*0) and* *R* *(0*,*1*,*0)*
*Mx < 0 and My < 0*	*L (−1*,*0*,*0) and L (0*,*1*,*0)*	*R* *(−1*,*0*,*0) and* *R* *(0*,*−1*,*0)*

*^1^* *L (1*,*0*,*0) and L (0*,*−1*,*0)* mean that the left arm rotates in a positive direction around the *X*-axis and negative direction around the *Y*-axis, respectively. *^2^ R* *(1*,*0*,*0) and* *R* *(0*,*1*,*0)* mean that the right arm rotates in a positive direction around the *X*-axis and the *Y*-axis, respectively.

**Table 3:** Parameters for single-arm operation and dual-arm operation.

Parameters	Single-Arm Operation	Dual-Arm Operation	
	Right Arm	Left Arm	Right Arm
Length of inserting step	10 mm	5 mm	5 mm
Length of searching step	5 mm	2.5 mm	2.5 mm
Speed of end-effector	40 mm/s	20 mm/s	20 mm/s
Rotation angle	1°	0.5°	0.5°

## Supplementary Materials

The following are available online at http://www.mdpi.com/1424-8220/17/9/2004/s1.
